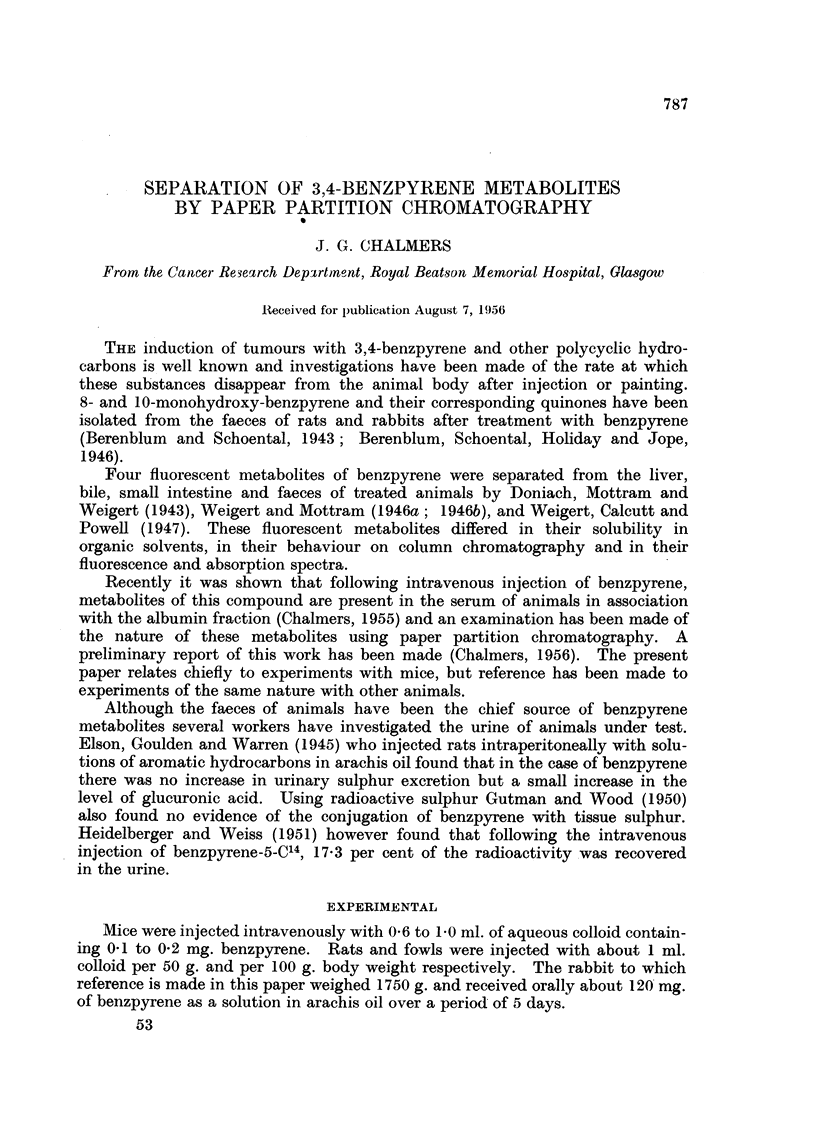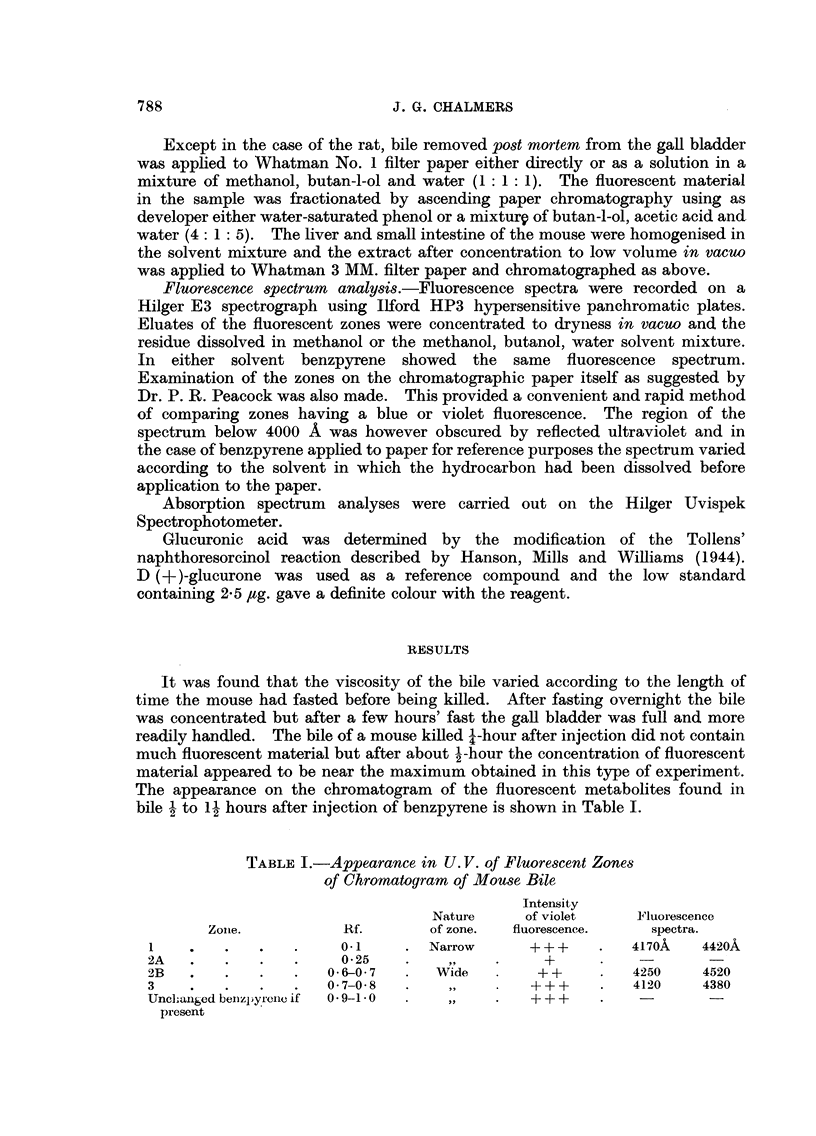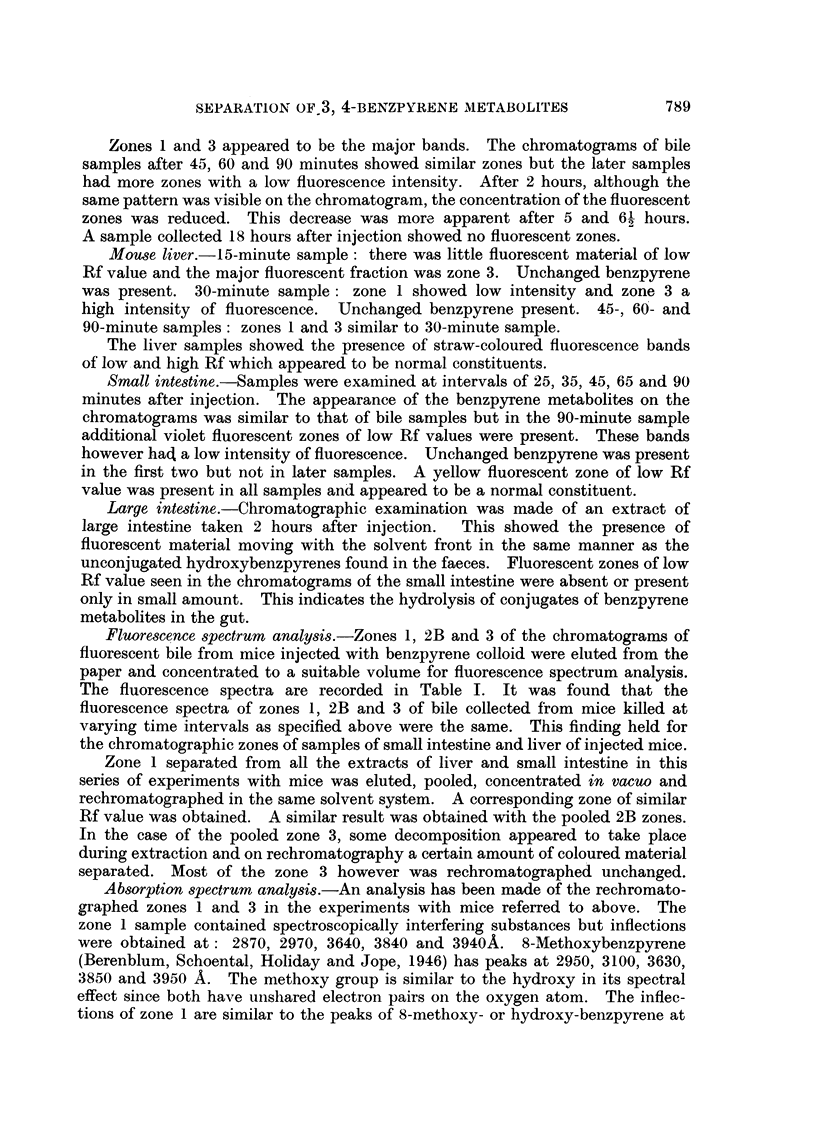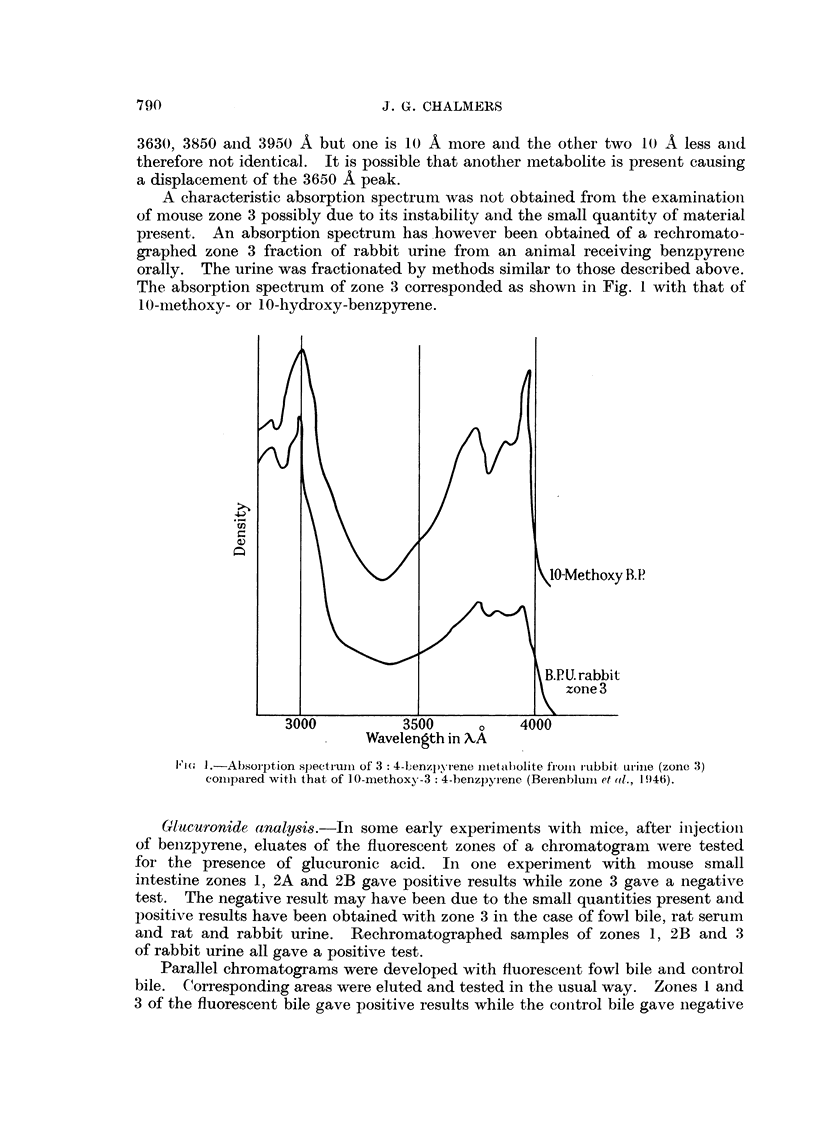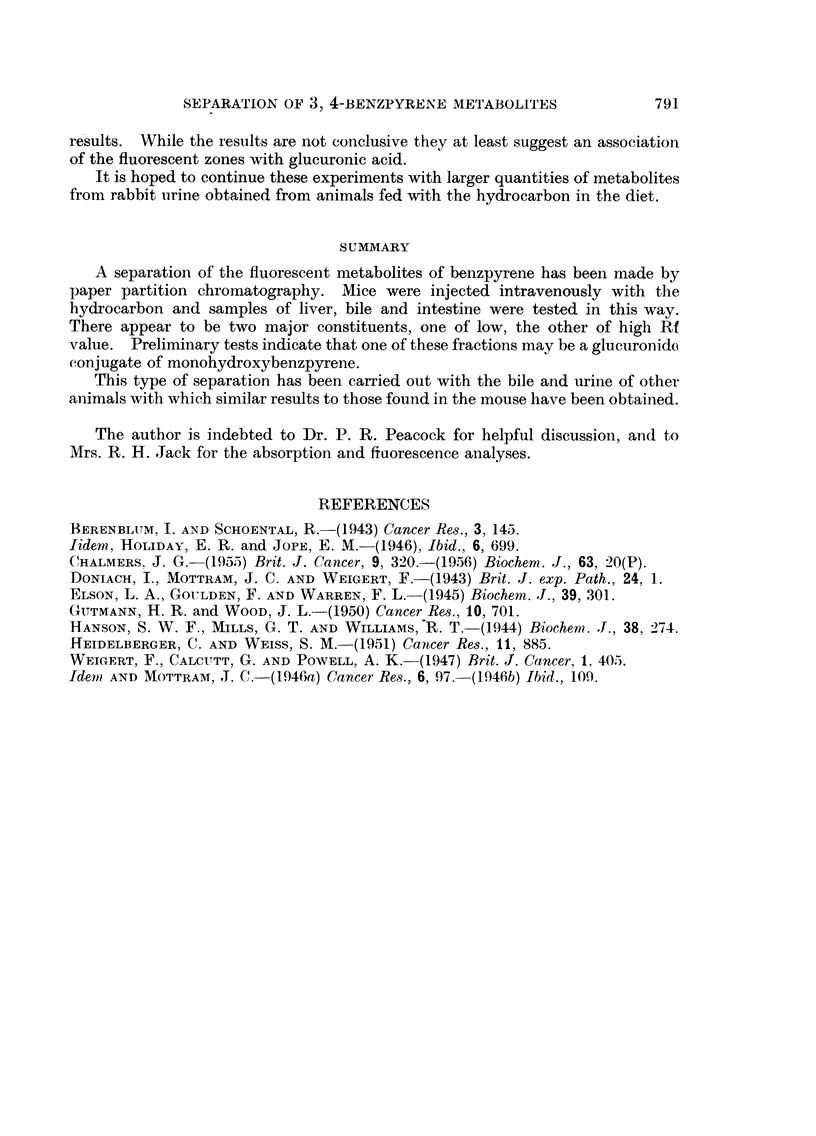# Separation of 3,4-Benzpyrene Metabolites by Paper Partition Chromatography

**DOI:** 10.1038/bjc.1956.95

**Published:** 1956-12

**Authors:** J. G. Chalmers


### SEPARATION OF 3,4-BENZPYRENE METABOLITES BY PAPER PARTITION CHROMATOGRAPHY

J. G. CHALMERS

*From the Cancer Research Department, Royal Beatson Memorial Hospital, Glasgow*

Received for publication August 7, 1956

THE induction of tumours with 3,4-benzpyrene and other polycyclic hydrocarbons is well known and investigations have been made of the rate at which these substances disappear from the animal body after injection or painting. 8- and 10-monohydroxy-benzpyrene and their corresponding quinones have been isolated from the faeces of rats and rabbits after treatment with benzpyrene (Berenblum and Schoental, 1943 ; Berenblum, Schoental, Holiday and Jope, 1946).

Four fluorescent metabolites of benzpyrene were separated from the liver, bile, small intestine and faeces of treated animals by Doniach, Mottram and Weigert (1943), Weigert and Mottram (1946*a* ; 1946*b*), and Weigert, Calcutt and Powell (1947). These fluorescent metabolites differed in their solubility in organic solvents, in their behaviour on column chromatography and in their fluorescence and absorption spectra.

Recently it was shown that following intravenous injection of benzpyrene, metabolites of this compound are present in the serum of animals in association with the albumin fraction (Chalmers, 1955) and an examination has been made of the nature of these metabolites using paper partition chromatography. A preliminary report of this work has been made (Chalmers, 1956). The present paper relates chiefly to experiments with mice, but reference has been made to experiments of the same nature with other animals.

Although the faeces of animals have been the chief source of benzpyrene metabolites several workers have investigated the urine of animals under test. Elson, Goulden and Warren (1945) who injected rats intraperitoneally with solutions of aromatic hydrocarbons in arachis oil found that in the case of benzpyrene there was no increase in urinary sulphur excretion but a small increase in the level of glucuronic acid. Using radioactive sulphur Gutman and Wood (1950) also found no evidence of the conjugation of benzpyrene with tissue sulphur. Heidelberger and Weiss (1951) however found that following the intravenous injection of benzpyrene-5-$C^{14}$, 17·3 per cent of the radioactivity was recovered in the urine.

### EXPERIMENTAL

Mice were injected intravenously with 0·6 to 1·0 ml. of aqueous colloid containing 0·1 to 0·2 mg. benzpyrene. Rats and fowls were injected with about 1 ml. colloid per 50 g. and per 100 g. body weight respectively. The rabbit to which reference is made in this paper weighed 1750 g. and received orally about 120 mg. of benzpyrene as a solution in arachis oil over a period of 5 days.

Except in the case of the rat, bile removed *post mortem* from the gall bladder was applied to Whatman No. 1 filter paper either directly or as a solution in a mixture of methanol, butan-l-ol and water (1 : 1 : 1). The fluorescent material in the sample was fractionated by ascending paper chromatography using as developer either water-saturated phenol or a mixture of butan-l-ol, acetic acid and water (4 : 1 : 5). The liver and small intestine of the mouse were homogenised in the solvent mixture and the extract after concentration to low volume *in vacuo* was applied to Whatman 3 MM. filter paper and chromatographed as above.

*Fluorescence spectrum analysis.*—Fluorescence spectra were recorded on a Hilger E3 spectrograph using Ilford HP3 hypersensitive panchromatic plates. Eluates of the fluorescent zones were concentrated to dryness *in vacuo* and the residue dissolved in methanol or the methanol, butanol, water solvent mixture. In either solvent benzpyrene showed the same fluorescence spectrum. Examination of the zones on the chromatographic paper itself as suggested by Dr. P. R. Peacock was also made. This provided a convenient and rapid method of comparing zones having a blue or violet fluorescence. The region of the spectrum below 4000 Å was however obscured by reflected ultraviolet and in the case of benzpyrene applied to paper for reference purposes the spectrum varied according to the solvent in which the hydrocarbon had been dissolved before application to the paper.

Absorption spectrum analyses were carried out on the Hilger Uvispek Spectrophotometer.

Glucuronic acid was determined by the modification of the Tollens' naphthoresorcinol reaction described by Hanson, Mills and Williams (1944). D (+)-glucurone was used as a reference compound and the low standard containing 2·5 $\mu$g. gave a definite colour with the reagent.

### RESULTS

It was found that the viscosity of the bile varied according to the length of time the mouse had fasted before being killed. After fasting overnight the bile was concentrated but after a few hours' fast the gall bladder was full and more readily handled. The bile of a mouse killed ¼-hour after injection did not contain much fluorescent material but after about ½-hour the concentration of fluorescent material appeared to be near the maximum obtained in this type of experiment. The appearance on the chromatogram of the fluorescent metabolites found in bile ½ to 1½ hours after injection of benzpyrene is shown in Table I.

TABLE I.—*Appearance in U.V. of Fluorescent Zones of Chromatogram of Mouse Bile*

| Zone. | Rf. | Nature of zone. | Intensity of violet fluorescence. | Fluorescence spectra. | |
|---|---|---|---|---|---|
| 1 | 0·1 | Narrow | +++ | 4170Å | 4420Å |
| 2A | 0·25 | ,, | + | — | — |
| 2B | 0·6–0·7 | Wide | ++ | 4250 | 4520 |
| 3 | 0·7–0·8 | ,, | +++ | 4120 | 4380 |
| Unchanged benzpyrene if present | 0·9–1·0 | ,, | +++ | — | — |

Zones 1 and 3 appeared to be the major bands. The chromatograms of bile samples after 45, 60 and 90 minutes showed similar zones but the later samples had more zones with a low fluorescence intensity. After 2 hours, although the same pattern was visible on the chromatogram, the concentration of the fluorescent zones was reduced. This decrease was more apparent after 5 and 6½ hours. A sample collected 18 hours after injection showed no fluorescent zones.

*Mouse liver.*—15-minute sample : there was little fluorescent material of low Rf value and the major fluorescent fraction was zone 3. Unchanged benzpyrene was present. 30-minute sample : zone 1 showed low intensity and zone 3 a high intensity of fluorescence. Unchanged benzpyrene present. 45-, 60- and 90-minute samples : zones 1 and 3 similar to 30-minute sample.

The liver samples showed the presence of straw-coloured fluorescence bands of low and high Rf which appeared to be normal constituents.

*Small intestine.*—Samples were examined at intervals of 25, 35, 45, 65 and 90 minutes after injection. The appearance of the benzpyrene metabolites on the chromatograms was similar to that of bile samples but in the 90-minute sample additional violet fluorescent zones of low Rf values were present. These bands however had a low intensity of fluorescence. Unchanged benzpyrene was present in the first two but not in later samples. A yellow fluorescent zone of low Rf value was present in all samples and appeared to be a normal constituent.

*Large intestine.*—Chromatographic examination was made of an extract of large intestine taken 2 hours after injection. This showed the presence of fluorescent material moving with the solvent front in the same manner as the unconjugated hydroxybenzpyrenes found in the faeces. Fluorescent zones of low Rf value seen in the chromatograms of the small intestine were absent or present only in small amount. This indicates the hydrolysis of conjugates of benzpyrene metabolites in the gut.

*Fluorescence spectrum analysis.*—Zones 1, 2B and 3 of the chromatograms of fluorescent bile from mice injected with benzpyrene colloid were eluted from the paper and concentrated to a suitable volume for fluorescence spectrum analysis. The fluorescence spectra are recorded in Table I. It was found that the fluorescence spectra of zones 1, 2B and 3 of bile collected from mice killed at varying time intervals as specified above were the same. This finding held for the chromatographic zones of samples of small intestine and liver of injected mice.

Zone 1 separated from all the extracts of liver and small intestine in this series of experiments with mice was eluted, pooled, concentrated *in vacuo* and rechromatographed in the same solvent system. A corresponding zone of similar Rf value was obtained. A similar result was obtained with the pooled 2B zones. In the case of the pooled zone 3, some decomposition appeared to take place during extraction and on rechromatography a certain amount of coloured material separated. Most of the zone 3 however was rechromatographed unchanged.

*Absorption spectrum analysis.*—An analysis has been made of the rechromatographed zones 1 and 3 in the experiments with mice referred to above. The zone 1 sample contained spectroscopically interfering substances but inflections were obtained at : 2870, 2970, 3640, 3840 and 3940Å. 8-Methoxybenzpyrene (Berenblum, Schoental, Holiday and Jope, 1946) has peaks at 2950, 3100, 3630, 3850 and 3950 Å. The methoxy group is similar to the hydroxy in its spectral effect since both have unshared electron pairs on the oxygen atom. The inflections of zone 1 are similar to the peaks of 8-methoxy- or hydroxy-benzpyrene at

3630, 3850 and 3950 Å but one is 10 Å more and the other two 10 Å less and therefore not identical. It is possible that another metabolite is present causing a displacement of the 3650 Å peak.

A characteristic absorption spectrum was not obtained from the examination of mouse zone 3 possibly due to its instability and the small quantity of material present. An absorption spectrum has however been obtained of a rechromatographed zone 3 fraction of rabbit urine from an animal receiving benzpyrene orally. The urine was fractionated by methods similar to those described above. The absorption spectrum of zone 3 corresponded as shown in Fig. 1 with that of 10-methoxy- or 10-hydroxy-benzpyrene.

FIG 1.—Absorption spectrum of 3 : 4-benzpyrene metabolite from rabbit urine (zone 3) compared with that of 10-methoxy-3 : 4-benzpyrene (Berenblum *et al.*, 1946).

*Glucuronide analysis.*—In some early experiments with mice, after injection of benzpyrene, eluates of the fluorescent zones of a chromatogram were tested for the presence of glucuronic acid. In one experiment with mouse small intestine zones 1, 2A and 2B gave positive results while zone 3 gave a negative test. The negative result may have been due to the small quantities present and positive results have been obtained with zone 3 in the case of fowl bile, rat serum and rat and rabbit urine. Rechromatographed samples of zones 1, 2B and 3 of rabbit urine all gave a positive test.

Parallel chromatograms were developed with fluorescent fowl bile and control bile. Corresponding areas were eluted and tested in the usual way. Zones 1 and 3 of the fluorescent bile gave positive results while the control bile gave negative results. While the results are not conclusive they at least suggest an association of the fluorescent zones with glucuronic acid.

It is hoped to continue these experiments with larger quantities of metabolites from rabbit urine obtained from animals fed with the hydrocarbon in the diet.

### SUMMARY

A separation of the fluorescent metabolites of benzpyrene has been made by paper partition chromatography. Mice were injected intravenously with the hydrocarbon and samples of liver, bile and intestine were tested in this way. There appear to be two major constituents, one of low, the other of high Rf value. Preliminary tests indicate that one of these fractions may be a glucuronide conjugate of monohydroxybenzpyrene.

This type of separation has been carried out with the bile and urine of other animals with which similar results to those found in the mouse have been obtained.

The author is indebted to Dr. P. R. Peacock for helpful discussion, and to Mrs. R. H. Jack for the absorption and fluorescence analyses.

### REFERENCES

BERENBLUM, I. AND SCHOENTAL, R.—(1943) *Cancer Res.*, **3**, 145.
*Iidem*, HOLIDAY, E. R. and JOPE, E. M.—(1946), *Ibid.*, **6**, 699.
CHALMERS, J. G.—(1955) *Brit. J. Cancer*, **9**, 320.—(1956) *Biochem. J.*, **63**, 20(P).
DONIACH, I., MOTTRAM, J. C. AND WEIGERT, F.—(1943) *Brit. J. exp. Path.*, **24**, 1.
ELSON, L. A., GOULDEN, F. AND WARREN, F. L.—(1945) *Biochem. J.*, **39**, 301.
GUTMANN, H. R. and WOOD, J. L.—(1950) *Cancer Res.*, **10**, 701.
HANSON, S. W. F., MILLS, G. T. AND WILLIAMS, R. T.—(1944) *Biochem. J.*, **38**, 274.
HEIDELBERGER, C. AND WEISS, S. M.—(1951) *Cancer Res.*, **11**, 885.
WEIGERT, F., CALCUTT, G. AND POWELL, A. K.—(1947) *Brit. J. Cancer*, **1**, 405.
*Idem* AND MOTTRAM, J. C.—(1946*a*) *Cancer Res.*, **6**, 97.—(1946*b*) *Ibid.*, 109.